# The impairment of speech perception in noise following pure tone hearing recovery in patients with sudden sensorineural hearing loss

**DOI:** 10.1038/s41598-021-03847-y

**Published:** 2022-01-17

**Authors:** Tongxiang Diao, Maoli Duan, Xin Ma, Jinjun Liu, Lisheng Yu, Yuanyuan Jing, Mengyuan Wang

**Affiliations:** 1 Department of Otolaryngology, Head and Neck Surgery, People’s Hospital, Peking University, Beijing, China; 2 School of Psychology, Beijing Normal University, Beijing, 100875 China; 3 Department of Clinical Science, Intervention and Technology, Karolinska Institute, Stockholm, Sweden; 4 Department of Otolaryngology Head and Neck Surgery & Audiology and Neurotology, Karolinska University Hospital, Karolinska Institute, 171 76 Stockholm, Sweden

**Keywords:** Psychology, Neurology

## Abstract

To explore whether patients with unilateral idiopathic sudden sensorineural hearing loss (uISSNHL) have normal speech in noise (SIN) perception under different masking conditions after complete recovery of pure tone audiometry. Eight completely recovered uISSNHL patients were enrolled in ISSNHL group, while 8 normal-hearing adults matched with age, gender, and education experience were selected as the control group. Each group was tested SIN under four masking conditions, including noise and speech maskings with and without spatial separation cues. For both ISSNHL and control groups a two-way ANOVA showed a statistically significant effect of masking type (p = 0.007 vs p = 0.012). A significant effect of perceived spatial separation (p < 0.001 vs p < 0.001). A significant interaction between masking type and perceived spatial separation was found (p < 0.001 vs p < 0.001). A paired sample T-test showed that the SIN perception of the control group was statistically significant lower than that of ISSNHL patients only under speech masking without spatial separation cues (p = 0.011). There were still abnormalities in the auditory center shortly after complete recovery in the ISSNHL group (within 2 weeks). However, the auditory periphery and higher-level ability to use spatial cues was normal.

## Introduction

Idiopathic sudden sensorineural hearing loss (ISSNHL) is defined as at least 30 dB sensorineural hearing loss in three sequential frequencies within 3 days with no identifiable cause^1^, which has an incidence ranging from 5 to 20 per 100,000 people in the Western countries and 19 per 100,000 in mainland China^2^. The pathogenesis of ISSNHL is still unknown, although a number of hypotheses have been proposed, including viral infection, vascular compromise, chronic inflammation, immunological diseases^3^, cochlear membrane rupture^4^, inner ear cell stress reaction^5^, hemorrhage of inner ear^6^, migraine^7^. Fortunately, about one-third of ISSNHL obtains complete recovery of hearing either spontaneously or after appropriate interventions^1,8^.

At present, the standard of hearing recovery of ISSNHL is mainly based on pure tone audiometry, however, the result of pure tone audiometry and speech recognition in quiet environment cannot fully reflect the auditory ability in daily life, which is full of reverberation and background noise^9^. Clinically, among patients with uISSNHL who have recovered completely (ISSNHL group) based on pure tone audiometry after treatment, some still complain of difficulty in speech perception in daily life. Previous studies on temporary hearing loss caused by noise exposure have found that despite the normal hair cell populations, temporary threshold shift caused by noise exposure can result in a permanent loss of low-spontaneous-rate auditory nerve fibers and reduction of auditory brainstem response wave-I amplitudes may affect SIN^10^. Although there have been some studies on the SIN ability of patients after sudden deafness^9,10^, to our knowledge, there have been no previous studies taking into account different mask conditions in the uISSNHL patients following complete recovery of pure tone hearing threshold.

SIN perception is a complicated multifaceted process. It is established that peripheral hearing loss, declined central auditory processing and impaired cognitive abilities are critical predictors to the deficit in understanding SIN^9,11^. Two types of maskers resulted in difficulties of SIN perception: noise masking and speech masking^12–14^. Noise masking is the difficulty when the auditory peripheral representation of the target sound is overwhelmed by competing sounds (when the masker are noise sounds and target is speech sound), originating primarily in the auditory periphery; speech masking refers to difficulty in the higher-order selective processing of audible target sounds due to perceptual/sensory similarities between the target and the masker (when both the target and masker are speech sounds), which was originated from the auditory central level. The role of higher-level cognitive abilities is important in speech recognition, the SIN perception performance is better when there is perceived spatial separation between targets and maskers. For example, the threshold of signal-to-noise ratios in speech perception is about 4–9 dB lower because of subjective 90-degree spatial separation (spatial release from masking, SRM)^12,13^. These results proved that SRM is one of the higher-level cognitive functions to improve speech intelligibility.

The present study is to investigate whether pure tone audiometry completely recovered uISSNHL patients have normal behavior in speech perception under noise masking and speech masking with or without using a perceived spatial separation paradigm, including noise and speech maskings with (noise masking’s separation condition and speech masking’s separation condition) or without spatial separation cues (noise masking’s colocation condition and speech masking’s colocation condition).

## Materials and methods

### Participants

Eight uISSNHL patients (6 females and 2 males, mean age = 44.9 years) met the following criteria were enrolled in the present study as the ISSNHL complete recovery (ISSNHL) group: (1) 18–65 years old; (2) acute unilateral SSNHL (≥ 30 dB) in three sequential frequencies of 0.25, 0.5, 1, 2, 3, 4, and 8 kHz compared to the healthy ear; (3) After treatment according to the Chinese sudden deafness guideline , 0.25, 0.5, 1, 2, 3, 4, and 8 kHz hearing defined as final hearing level better than 25 dB (6, /8) or reached healthy side hearing level (2/8)^15^; (4) Patients with middle ear disease, retro cochlear disorders, and congenital deafness had been excluded. The clinical characteristics of the ISSNHL group are shown in Table 1. In contrast, eight normal hearing adults (pure-tone threshold less than 25 dB between 0.25 and 8 kHz) matched age, gender, and education experience with each patient as control group to control the working memory differences of these two groups.

**Table 1:** Clinical characteristics of ISSNHL group.

Clinical characteristics	ISSNHL group
N. of patients	8
Age (years)	45.00 ± 11.71
Lateral (left vs. right)	2/6
Sex (male vs. female)	2/6
Onset—therapy delay (day)	4.63 ± 3.66
Onset-complete recovery (day)	12.63 ± 2.72
Duration of CR to test (day)	3.75 ± 4.17
PTA (affected side dB)	73.44 ± 25.01
PTA (health side dB)	21.41 ± 4.88
PTA (discharge dB)	23.59 ± 4.14
Recovery (dB)	49.83 ± 23.68

*CR* complete recovery.

### Assessment of peripheral hearing

Audiometric measurements were obtained following the procedure recommended by the Chinese Acoustics Standardization Committee, using a Astera (Natus, UK) Unity PC audiometer transducer in a sound-attenuating booth. Hearing thresholds had been obtained for air and bone conduction at 0.25, 0.5, 1, 2, 3, 4, and 8 kHz Separately. Pure-tone audiometry had been performed every 5–7 days. The patients participated the study within two weeks when their pure-tone audiometry recovered back to normal hearing (≤ 25 dB) or reached healthy side hearing level.

### Study design

A set of special Chinese nonsense sentences^13,16^ were used as target ones which were translated from English nonsense sentences developed by Helfer^12,16–19^. For instance, the English translation of a Chinese nonsense sentence “一些条令已经翻译我的大衣” is “Some *rules* had *translated* my *coat*” (the three 2-character keywords are italic)^17^. Target sentences with a naturally stable rate were spoken by a young female talker (talker A) at an average rate of 5.4 syllables/s (with the standard deviation of 0.7 syllables/s), while the duration of a sentence was about 2–3 s. The noise masker was a stream of steady-state speech-spectrum noise, whose spectrum was representative of the average spectrum of target sentences. The speech masker was a 47-s loop of digitally combined continuous recordings for Chinese nonsense sentences, spoken by two different young female talkers (talkers B and C).

Patients from the ISSNHL group and control group were tested bilateral (left, right) SIN perception of various signal-to-noise ratios (SNRs) under speech and noise masking conditions. Each participant has to complete four consequent levels of SNRs (− 12, − 8, − 4, 0 dB for most of control group, − 8, − 4, 0, 4 dB for ISSNHL group). In addition, the benefits of spatial separation cues under these two types of masking were analyzed. Namely, the experiment had four within-subject factors: masker type (noise masker, speech masker), the side from which participants perceived target (left, right), perceived spatial separation (colocation, separation) and SNRs (four consequent levels from − 12, − 8, − 4, 0, 4 dB). Besides, there was a between-subject factor: group (ISSNHL patients, matched normal hearing adults). Fifteen target sentences were used in each condition, and 480 trials totally were used for each participant.

### Ethics approval

This cohort research was approved by the Peking University People’s Hospital Research Ethics Committee (Beijing, China) (2021PHB147-001). Written informed consent for participating this experiment was received from all participants (including control group as well). And all methods were performed in accordance with the relevant guidelines and regulations.

### Data analysis

A logistic psychometric function (Eq. (1)) was fit to the mean data across the four SNR levels for each participant, where y is the probability of correct identification of keywords, x is the SNR corresponding to y, μ is the SNR corresponding to 50% correct on the psychometric function, and σ determines the slope of the psychometric function:

1 $$\text{y}=\frac{1}{1+{\text{e}}^{-\upsigma (\text{x}-\upmu )}}.$$

The release amount of spatial unmasking was calculated as the difference in the threshold between separation and colocation, for the noise masker and speech masker separately. Larger spatial release amount indicates larger cognitive benefit from spatial attention and better binaural auditory processing to the target in perceiving SIN.

### Statistical analysis

SPSS software (ver. 20.0; SPSS Inc., Chicago, IL, USA) was used for statistical analysis. Categorical variables were analyzed by the chi-squared test. Binary logistic regression models were further to explore the results of univariate analyses. In all analyses, p < 0.05 was considered as statistical significance.

## Results

### Clinical and epidemiological characteristics of the ISSNHL group

8 uISSNH patients’ clinical and epidemiological characteristics are shown in Table 1, with an average age of 45.00 ± 11.71 years old. Among all the 8 cases, 2 patients on the left side and 6 patients on the right side. There were 2 males and 6 females. The average duration from onset to treatment was 4.63 ± 3.66 days, the average duration from onset to recovery was 12.63 ± 2.72 days, and the average duration from recovery to testing was 3.75 ± 4.17 days. The average hearing threshold of the affected ear at admission was 73.44 ± 25.01 dB, while the average hearing threshold at discharge was 23.59 ± 4.14 dB.

### The influence of target signals from different locations on the speech perception

**Figure 1:**
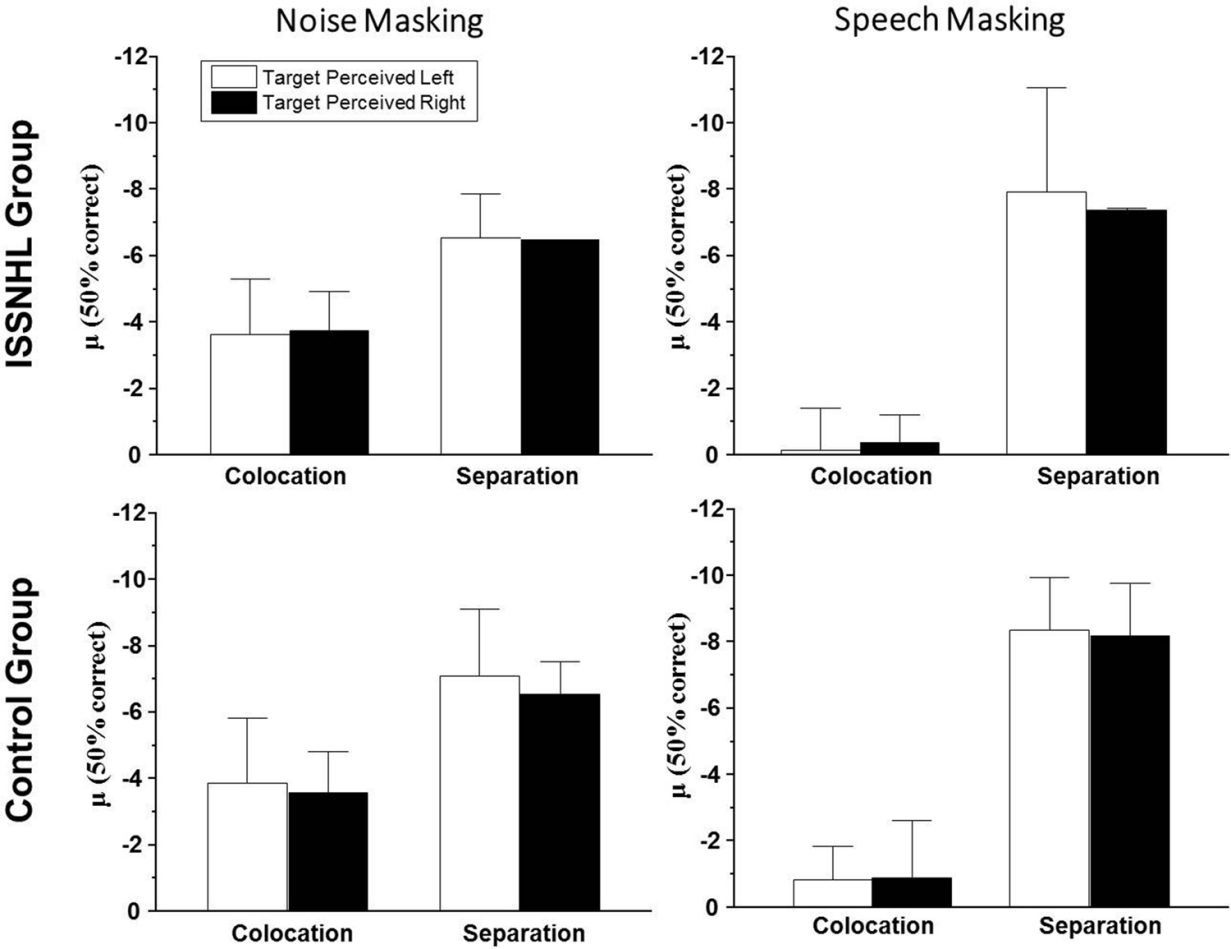
Participants’ SIN perception threshold μ when targets were perceived from left to the threshold when targets were perceived from right.

In view of the situation of unilateral hearing loss in ISSNHL patients, there were two kinds of perceived target speech locations, left and right. This within-subject factor can help us to pursue whether the SIN perception ability of the uISSNHL patients has lateral bias. The mean SINs threshold (μ) of two groups under each condition was calculated as the Fig. 1. The results showed that there were no significant differences of SIN perception threshold μ between two sides in both two groups no matter under speech masking or noise masking. So, we recalculated μ in the other factors based on both sides, in order to simplify follow-up analysis and clarify the integral performance in SIN perception test.

### SIN perception and SRM in ISSNHL group

**Figure 2:**
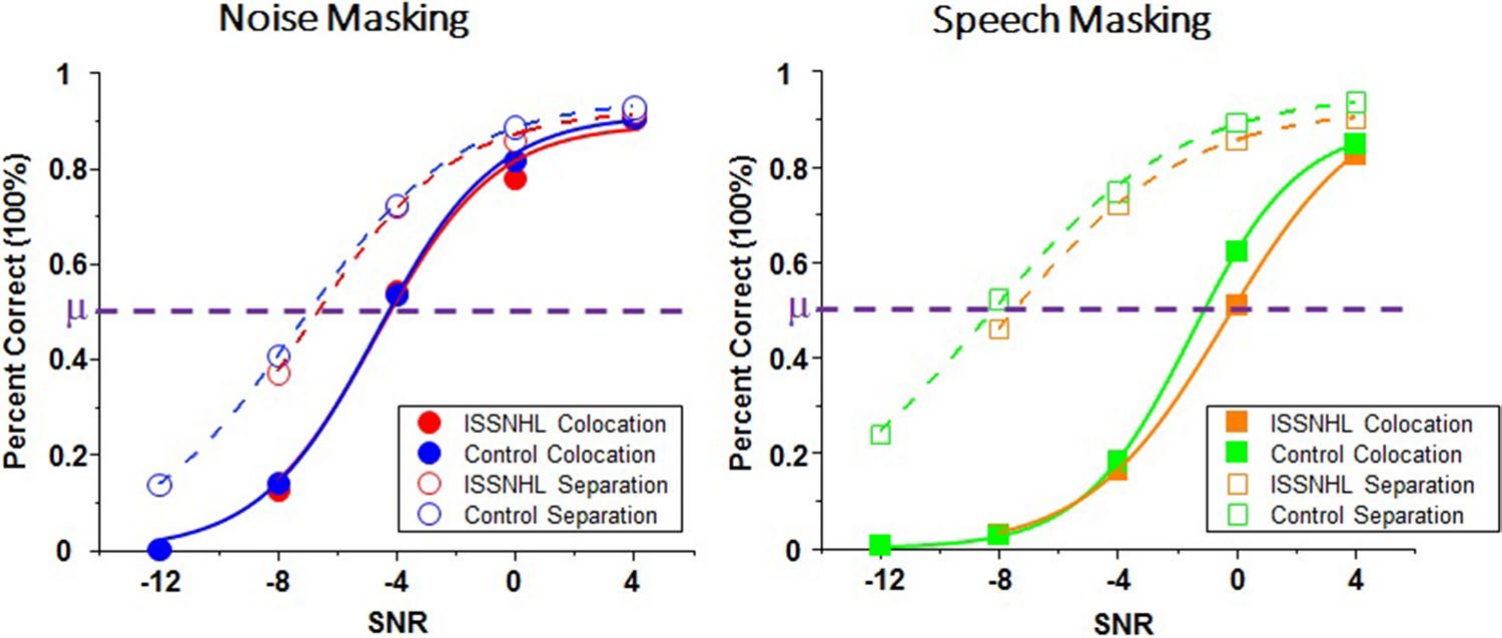
Group mean percent of correct as a function of signal-to-noise ratio (SNR) in noise masking condition (left panel) and speech masking condition (right panel).

**Figure 3:**
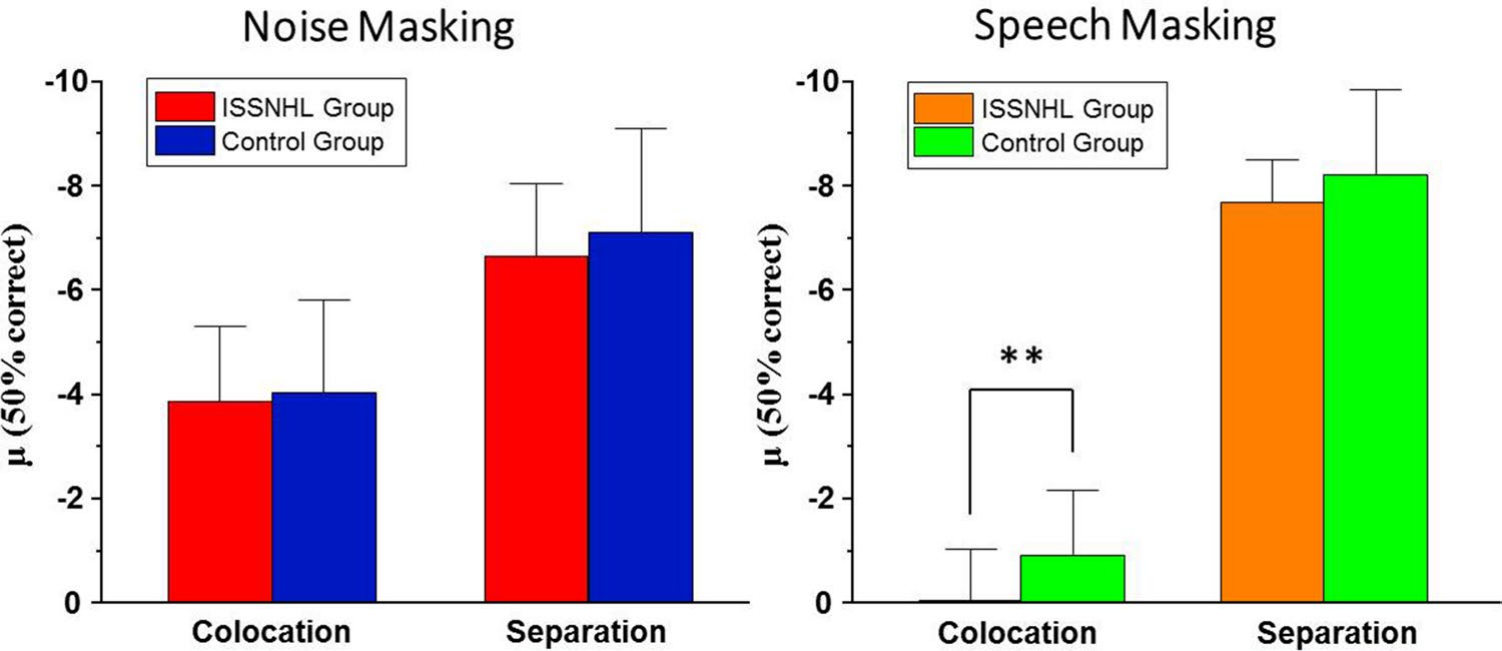
SIN perception threshold under each condition. The error bars represent the standard error of the mean (SEM).

For ISSNHL patients, a two-way ANOVA showed a statistically significant effect of masking type (F = 8.39, p = 0.007), a significant effect of perceived spatial separation (F = 118.41, p < 0.001), a statistically significant interaction between masking type and perceived spatial separation (F = 25.63, p < 0.001) shown in Fig. 2. The measure to calculate the effect of spatial release from masking (SRM, Δμ) is the SIN perception threshold under colocation condition minus the threshold under separation. As Fig. 3 showed, the ISSNHL group performed spatial release from masking (in other words, Δμ > 0) under noise masking (M = 2.79 dB, SD = 0.85 dB, t = 9.30, p < 0.001) and speech masking (M = 7.65 dB, SD = 1.68 dB, t = 12.91, p < 0.001).

### SIN perception and SRM in control group

For control group, a repeated measures analysis of variance ANOVA showed a significant main effect of masking type (F = 7.29, p = 0.012), a statistically significant effect of perceived spatial separation (F = 194.17, p < 0.001), a significant interaction between masking type and perceived spatial separation (F = 32.18, p < 0.001) as show in Fig. 2. As for the SRM, the control group also performed spatial release from masking (in other words, Δμ > 0) under noise masking (M = 3.08 dB, SD = 0.34 dB, t = 25.50, p < 0.001) and speech masking (M = 7.82 dB, SD = 0.90 dB, t = 23.06, p < 0.001) (Fig. 3).

### Group differences in SIN perception and SRM

Figure 2 shows the group mean percent of correct as a function of SNR and the SIN perception threshold (μ, in dB) computed by the psychometric function under two types of masking conditions, noise masking and speech masking. According to the Fig. 3, a paired sample T test showed that the SIN perception threshold μ of control group was statistically significantly lower than that of ISSNHL patients only under speech masking’s colocation conditions (t = 3.46, p = 0.011), but neither noise-masking (colocation: t = 0.32, p = 0.757; separation: t = 0.74, p = 0.481) nor speech masking’s separation conditions (t = 0.75, p = 0.477). It showed that patients performed significantly worse than normal hearing control under speech masking without spatial separation cues and performed as well as the control group under the other conditions.

**Figure 4:**
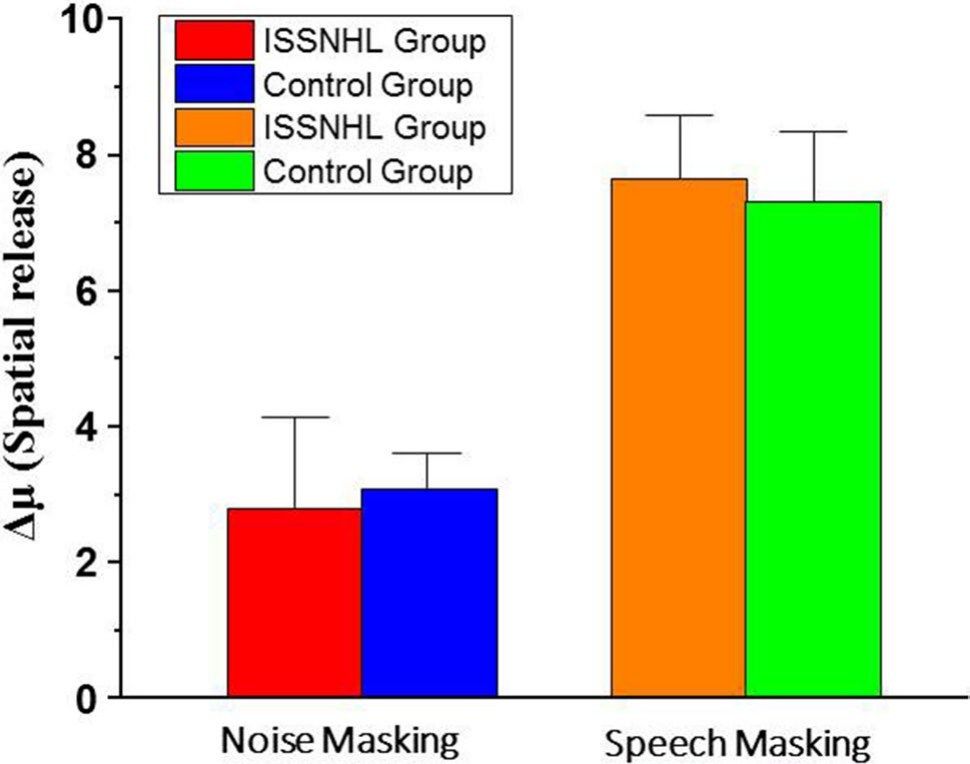
The spatial release from masking of two groups under noise masking and speech masking. The error bars represent the standard error of the mean (SEM).

Besides, a paired sample T test showed that there were no significant differences of SRM between ISSNHL patients and control under noise masking (t =  − 0.823, p = 0.438) or speech masking (t = 0.436, p = 0.676). Moreover, the effect of spatial release from speech masking was statistically significantly higher than that from noise masking for ISSNHL patients (t = 9.38, p < 0.001), just as well as control group (t = 11.38, p < 0.001). These results indicate that ISSNHL patients could benefit from spatial separation cues, especially under speech masking, and their ability of spatial release was the same as their normal hearing peers (Fig. 4).

## Discussion

Our results indicated that patients of ISSNHL group performed statistically significantly worse than the control group only under speech masking without spatial separation, but there are no remarkable differences between these two groups under other masking conditions. Furthermore, patients of ISSNHL group could benefit from spatial separation cues, especially under speech masking, and their ability of spatial release was the same as the control group.

### There was no difference in the SIN perception between ISSNHL and control group under noise masking

Noise masking which is interference produced in the spectrum of noise, originating primarily in the auditory periphery. The cochlea is still considered to be the most probable lesion site of sudden deafness^20^. The effects of outer hair cell (OHC) loss on hearing have been well described in several species across a number of studies, as it can result in increased hearing thresholds of associated frequencies and decline of frequency selectivity. In contrast, damage to inner hair cells (IHCs) and auditory nerve fibers are difficult to be detected, for they can only affect the threshold sensitivity when exceeding 80% are damaged. Fortunately, the SIN perception tests are sensitive to moderate to severe IHCs’ damage, so in this study, it was used to test the function of IHCs^21^. In this study, there was no statistically significant difference in SIN perception between these two groups under noise masking, suggesting that the hair cell function of the ISSNHL group was similar with that of the control group. Although some studies have shown that there may be hidden loss of hair cells that cannot be recognized by psychoacoustic tasks^22^, in this study, it is illustrated that the inner ear function of the ISSNHL group was equivalent to that of the control group, which suggested that the function of auditory periphery had recovered completely.

### ISSNHL group performed significantly worse than control group under speech masking without spatial separation

In previous research, sentences with no context or sense have been widely used to investigate different groups of people’s speech perception. Wilson et al.^23^ found that a measure named QuickSIN (Quick Speech in Noise test) that syntactically correct sentences with low semantic cues, is more sensitive to performance difference between normal hearing and hearing impaired groups than the BKB-SIN (Bamford–Kowal–Bench SIN) and Hearing In Noise Test (HINT), which use meaningful sentences. Thus, as a more sensitive measurement, the nonsense sentences in Mandarin Chinese which is described in detail in part method were used in current SIN perception research.

It has been documented that when the target signal and the masking signal are both speech signals, because the speech masking signal can also activate the speech system related to speech recognition and comprehension, it will interfere with the recognition of the target signal. It is suggested that speech masking starts from the auditory central level^12,13^. The damage of sensory system can ultimately lead to changes in neural representation via injury-induced plasticity. In the auditory system, restricted damage to the peripheral hearing can lead to robust plastic changes in the auditory cortex. A large number of studies have described the pattern and time course of plasticity in various cortical and brainstem regions caused by unilateral deafness. That is, even if there is no permanent change in the peripheral hearing threshold, the neural activity of the auditory cortex can be changed^24–26^. The neurophysiological evidence of functional reorganization of the central auditory pathway in patients with acute uISSNHL can be found in the very early central processing of transient sounds arriving at the auditory cortex^20^. The results of this study also suggested that although pure tone audiometry and peripheral auditory function completely recovered, the changes of central auditory function caused by the sudden deafness could not be fully restored in a short period (2 weeks).

### The spatial release ability of ISSNHL group was the same as the control group

In the process of speech masking, the cognitive processing systems need to use some certain clues, including the visual information such as lip shape and body posture, familiarity with the spatial position and the contents of the speech, and other related information like voice are all related to this cognitive process. It has been well documented that spatially separating the target signal from the masker can improve the recognition of the target signal^12,13^. The performance of speech perception in different types of masking and the ability that separating target signal from masking by using spatial separation cues reflect the central auditory processing and cognitive function. After unilateral hearing loss, learning-induced changes in adult tonotopic map plasticity are traditionally thought to require the precisely-timed involvement of neuromodulators recruited by arousal or attention. In this study, there was no difference in the ability of using spatial cues between the two groups, namely there was no difference in the higher cognitive function.

### Limitations

First, longitudinal follow-up can determine whether the central changes of these patients can be improved and whether intervention is needed, which is of greater clinical importance. Secondly, further electrophysiological studies, such as frequency following response (FFR) or auditory brainstem response(ABR), can be carried out on the patients in the follow-up to locate the lesion site of central auditory system. Finally, although there are some deficiencies, the preliminary exploration of this paper confirmed that patients who have complete recovery of pure tone audiometry after uISSNHL do have difficulties in SIN perception, which is worthy of attention, and also provides scientific basis for the treatment of unilateral sudden deafness. In addition, our study sample is relatively small and further study is needed in the future.

## Conclusions

This study is to explore the SIN perception among uISSNHL patients after complete recovery of pure tone audiometry under different types of masking conditions. The results of this study indicated that with peripheral auditory function completely recovery, there were still some abnormalities in the function of central auditory system, resulting in the impairment of speech perception under speech masking. We speculated that it may be related to the central compensation induced by unilateral sudden deafness, and further follow-up is needed. However, the higher cognitive function of using spatial cues of the ISSNHL group was the normal as the control group.

## Data Availability

Data will be shared by request from any qualified investigator. All authors had access to the final study data and material.
